# FROM CELLS TO SYSTEMS

**DOI:** 10.1093/geroni/igad104.1473

**Published:** 2023-12-21

**Authors:** Raya Kheirbek, Kenzie Latham-Mintus

## Abstract

Individuals who have been incarcerated may experience chronic pain due to various reasons, such as physical injuries sustained during their time in prison, untreated chronic medical conditions, or addiction to prescription medications. The pain may persist into late life, resulting in reduced quality of life, increased healthcare costs, and limited access to end of life care and hospice services. This issue requires collaboration among experts in pain management, geriatrics, and palliative care to develop effective interventions that address the unique needs of this population. Suicide among veterans is another pressing issue that requires an interdisciplinary approach. Incarcerated veterans may face numerous challenges upon release, including limited access to healthcare and social support, economic instability, and the stigma associated with a criminal record. These factors may contribute to increased risk of suicide. Addressing this issue requires a collaborative effort among experts in mental health, social work, and criminal justice to develop comprehensive programs that provide support for veterans transitioning from incarceration to civilian life. Being imprisoned can have lasting negative health consequences that persist throughout a person’s lifetime. Incarcerated individuals may face limited access to quality end-of-life care and hospice services, resulting in increased suffering during their final days. The experience of incarceration can also lead to social isolation and stigma, exacerbating mental health issues and further decreasing the quality of life at the end of life. Effective interventions are needed to address the unique healthcare needs of incarcerated individuals, particularly as they approach the end of life

